# Reconstruction of Saccular and Dissected Intracranial Aneurysms Using Solitaire™ AB Stents

**DOI:** 10.1371/journal.pone.0057253

**Published:** 2013-02-26

**Authors:** Kai-Jun Zhao, Yong-Wei Zhang, Yi Xu, Bo Hong, Qing-Hai Huang, Wen-Yuan Zhao, Peng-Fei Yang, Jian-Min Liu

**Affiliations:** 1 Department of Neurosurgery, Changhai Hospital, Second Military Medical University, Shanghai, China; 2 Department of Neurology & Neurosurgery, Changhai Hospital, Second Military Medical University, Shanghai, China; University of Münster, Germany

## Abstract

**Introduction:**

We aimed to evaluate the feasibility, safety, efficacy, and predictors for outcome of reconstructive treatment with Solitaire™ AB stent(s) based on 54 cases of saccular aneurysms and 14 of acute symptomatic dissecting aneurysms.

**Methods:**

Fifty-eight consecutive patients (M/F = 28/30; median age, 53 years) harbouring 68 aneurysms (ruptured/unruptured = 12/56) underwent treatment with Solitaire™ AB stent(s) implantation between April 2010 and August 2011 in our institution. The data were retrospectively reviewed and analysed.

**Results:**

The technical success rate of Solitaire™ AB stenting was 100%. The rates of the overall and the treatment-related adverse events were 9% (6/68) and 6% (4/68), respectively, and the recurrent rate was 1% (1/68). All of the adverse events (n = 6) occurred in tiny (n = 1, ≤3 mm) or small (n = 5, >3 to ≤10 mm) aneurysms. The majority (75%, 3/4) of thromboembolic events (thrombus, n = 2; infarction, n = 2) occurred in ruptured lesions, and 2 intraprocedural aneurysm ruptures occurred in the course of coiling when the stent(s) was/were applied within 6 months. Subarachnoid haemorrhages (SAH, *p<0.05*) and immediate occlusion grades (*p<0.05*) were predictors for overall adverse events by univariate analysis. Compared with the immediate post-treatment angiographic results, the follow-up angiographic imaging (mean, 13 months; range, 6–25 months) revealed that stent(s) implantation enhanced the rate of class I occlusion from 34% (23/68) to 93% (63/68). SAH was the only predictor for unfavourable outcomes (the modified Rankin Scale score [mRS], 2–6) during the mean 19-month (range, 12–27 months) of clinical follow-ups (*p<*0.05).

**Conclusions:**

Although the complete obliteration of tiny and small aneurysms without complications remains a challenge, stent(s) implantation could lead to further occlusion of incompletely coiled aneurysms. SAH and the occlusion grade were the primary predictors for adverse events. SAH was the only predictor for unfavourable outcomes by univariate analysis.

## Introduction

Endovascular coiling has emerged as the first-line treatment option for wide-necked intracranial aneurysms [1], [2]. For certain ruptured or unruptured wide-necked aneurysms, the appropriate adjunctive tools are crucial [3]. Compared with balloon-assisted coiling, stent-assisted coiling can provide a “scaffolding” for denser packing, reducing the risks of recurrence and rebleeding. The use of stenting alone has also been well applied for tiny [4] and fusiform aneurysms [5]. The multiple stents overlapping technique [6], [7] is also an alternative for the reconstruction of dissecting aneurysms in acute cases, such as SAH, acute posterior circulation stroke or neck pain.

Recently, 58 suitable patients harbouring 68 aneurysms underwent individualised treatment with a new stent (“Solitaire™ AB”) and the forgoing techniques in our institution. Although several small studies have provided preliminary results regarding the use of Solitaire™ stents for saccular aneurysms [8], [9], the predictors for unfavourable outcomes (mRS, 2–6) [10] related to Solitaire™ have not been fully elucidated. Moreover, little is known of the Solitaire™ AB stent results for the treatment of acute symptomatic dissecting aneurysms. Additionally, stenting-only with Solitaire™ stent(s) for the treatment of saccular or dissecting lesions has not been reported. Although the aneurysms in this study were of different aetiologies, they were commonly analysed since they were all suitable for endovascular reconstruction with Solitaire™ AB stent(s).

## Materials and Methods

This retrospective study was approved by our institution’s ethics committee.

### Patients

A retrospective review was conducted on the inpatient and outpatient angiographic and clinical charts to identify cases in our institution treated with Solitaire™ AB (Covidien/ev3) stent implantation between April 2010 and August 2011. The exclusion criteria included: (1) cases treated with different types of stents (n = 3); (2) cases lost to follow-up (n = 4). A total of 58 consecutive patients (M/F = 28/30; median age, 53 years; range, 17–77 years) harbouring 68 intracranial aneurysms (Hunt-Hess grade: 0–II in 57, IV in 1) were identified and enrolled in this study.

### Aneurysms

The dissecting aneurysms were diagnosed when fusiform or irregular dilatations of the intradural artery trunk were revealed by vascular imaging. A total of 52 saccular aneurysms (ruptured/unruptured = 9/43) and 3 unruptured symptomatic internal carotid artery dissection aneurysms (ICADAs) were located in the anterior circulation. The remaining 2 unruptured saccular aneurysms and 11 symptomatic intradural vertebral artery dissection aneurysms (VADAs, ruptured/unruptured = 3/8) were located in the posterior circulation, including 3 posterior inferior cerebellar artery (PICA)-involving lesions (ruptured/unruptured = 1/2). According to the morphology as seen by angiographic imaging, this series included 6 fusiform dissecting and 8 lateral protrusion-shaped dissecting aneurysms. Of the 68 lesions, 18 were designated as tiny (≤3 mm), 47 as small (>3 to ≦10 mm), and 3 as large (>10 to ≦25 mm) based on their maximum diameters. All the saccular and lateral protrusion-shaped dissecting aneurysms (n = 8) were wide-necked (neck>4 mm or dome/neck ratio<2), and the 6 fusiform aneurysms had no defined neck.

### Procedures

Two hours prior to the procedure, the patients with acutely ruptured aneurysms were preloaded with 300 mg clopidogrel and 300 mg aspirin. All the patients with unruptured aneurysms were preloaded daily with clopidogrel (75 mg) and aspirin (100 mg) for 3 days prior to the procedure. All the patients received systemic intravenous heparin after placement of the femoral sheath for an activated clotting time between 250–300 seconds. All the patients were treated under general anaesthesia by experienced interventional neurosurgeons. The stent placement was defined as a successful implantation if the single stent, or the multiple telescopically overlapped stents, extended at least 5 mm past each side of the aneurysm neck (saccular and lateral protrusion-shaped dissection aneurysms) or past the border of the dissection lesions (fusiform-shaped) of the target vessel. Post-procedure, the patients were kept on a 6-week dural antiplatelet regimen of 75 mg Plavix and 100 mg aspirin daily, followed by 100 mg aspirin indefinitely.

### Follow-ups

Angiographic control follow-ups were performed with conventional angiography at 6 months, 12 months, and annually thereafter. For fusiform dissecting aneurysms, the immediate and follow-up occlusion grades were defined as complete obliteration (class I), near-complete obliteration (class II), and partial obliteration (class III) [11]. For saccular and lateral protrusion-shaped dissecting aneurysms, the occlusion grade was defined as complete occlusion (class I), residual neck (class II), and residual aneurysm (class III) based on the Raymond classification [12]. For the cases treated by stenting alone, the residual contrast time within the aneurysm was increased moderately after the stent(s) implantation, and this was also regarded as a class III occlusion. The angiographic results were interpreted independently by 3 authors (Jian-Min Liu, Qing-Hai Huang and Wen-Yuan Zhao, each with 8–12 years of neurointerventional experience) using the Raymond classification. When there was no filling of contrast medium, a substantial shrink of contrast flow, no change of contrast flow, or an increase in contrast flow compared to the control angiogram performed immediately after treatment, the respective diagnoses of angiographic cure, improvement, stability, or recurrence were made. The mRS score acquired via neurologic examination or a telephone interview [13] at the most recent clinical follow-up was considered to be the final outcome and was categorized into two groups: favourable (mRS, 0–1) and unfavourable outcomes (mRS, 2–6) [10]. 10 of the 58 patients had 2 unruptured aneurysms which were independently treated, resulting in a total of 68 treated lesions with clinical follow-up results. All the follow-up results were summarised and analysed in July 2012.

### Statistical Analysis

Statistical analysis was performed using the SPSS software package (SPSS 17.0) to evaluate whether the univariate variables, including patient demographics and angiographic features of the aneurysms, had an impact on adverse events or unfavourable outcomes. The categorical and dichotomous variables were examined using Pearson’s *x* ^2^ test or Fisher’s exact test, as appropriate. A *p* value<0.05 was considered statistically significant.

## Results

### Treatment and Angiographic Results

The baseline characteristics of the 68 lesions are shown in Tables 1 and 2. The angiographic follow-ups (n = 68) were available for a mean of 13 months (range, 6–25 months). All the embedded stents were successfully performed, and the technical success rate of the stent placement was 100%. In the early stages (within 6 months) of the Solitaire™ AB stent application, 2 acute in-stent thrombi, 2 intraprocedural aneurysm ruptures, and 2 postprocedural cerebral infarctions were encountered. The rates of the overall and treatment-related adverse events were 9% (6/68) and 6% (4/68), respectively.

**Table 1:** The Immediate and Follow-up Angiographic Results of 68 Lesions Reconstructed with Different Methods.

Variables	Lesions	Immediate Results	Follow-up Results	Total
**Single stent with coils**				
Yes	ICADAs	Class II	Cured	2
Yes	Saccular	Class I	Cured	23
Yes	Saccular	Class II	Cured	11
Yes	Saccular	Class III	Cured	13
Yes	Saccular	Class III	Improved	1
**Total**				**50**
**Stenting-only**				
Single stent	Saccular (Tiny)	Class III	Unchanged	3
Multiple stents	VADAs	Class III	Cured	3
**Total**				**6**
**Multiple stents with coils**				
Yes	VADAs	Class III	Cured	7
Yes	VADA	Class III	Recurrence	1
Yes	ICADA	Class III	Cured	1
Yes	Saccular	Class III	Cured	2
Yes	Saccular	Class II	Cured	1
**Total**				**12**

ICADA(s), internal carotid artery dissection aneurysm(s); VADA(s), vertebral artery dissection aneurysm(s).

**Table 2:** Impact of Patient Demographics (n = 58) and Aneurysm Characteristics (n = 68) on Adverse Events.

Variables	n	Thromboembolism(n = 4)			Rupture(n = 2)			Overall Adverse Events(n = 6)		
		Yes	No	*p*	Yes	No	*p*	Yes	No	*p*
**Patient Sex, no. (%)**				*0.66*			*1.00*			*0.73*
Male	28	1 (4)	27 (96)		1(4)	27(96)		2 (7)	26 (93)	
Female	30	3 (10)	27 (90)		1 (3)	29(97)		4 (13)	26 (87)	
**SAH, no. (%)**				**0.02**			0.32			***0.006***
Ruptured	12	3 (25)	9 (75)		1 (8)	11(92)		4 (33)	8 (67)	
Unruptured	56	1 (2)	55 (98)		1 (2)	55(98)		2 (4)	54(96)	
**Aneurysm location, no. (%)**				1.00			1.00			*0.27*
Anterior circulation	55	4 (7)	51 (93)		2 (4)	53(96)		9 (16)	46(84)	
Posterior circulation	13	0 (0)	13 (100)		0 (0)	13(100)		0 (0)	13(100)	
**Aneurysm shape, no. (%)**				0.57			1.00			*0.23*
Saccular aneurysm	54	4 (7)	50 (93)		2 (4)	52(96)		9 (17)	45(83)	
Dissection aneurysm	14	0 (0)	14 (100)		0 (0)	14(100)		0 (0)	14(100)	
**Aneurysm size, no. (%)**				0.90			0.63			0.73
Tiny	18	1 (6)	17 (94)		0 (0)	18(100)		2 (11)	16(89)	
Small	47	3 (6)	44 (94)		2 (4)	45(96)		7 (15)	40(85)	
Large	3	0 (0)	3 (100)		0 (0)	3(100)		0 (0)	3(100)	
**Stent Strategy**				0.57			1.00			*0.39*
Single stent with or without coiling	53	4 (8)	49 (92)		2 (4)	51(96)		6 (11)	47 (89)	
Multiple stents with or without coiling	15	0 (0)	15 (100)		0 (0)	15(100)		0 (0)	15(100)	
**Stent Deployment Technique**										
Stenting before coiling (mesh technique)	5	0 (0)	5 (100)	0.66	0 (0)	5 (100)	0.82	0 (0)	5 (100)	0.53
Stenting after coiling (jailing or semi-jailing)	57	4 (7)	53 (93)		2 (4)	55 (96)		6 (11)	51 (89)	
Stenting-only	6	0 (0)	6 (100)		0 (0)	6 (100)		0 (0)	6 (100)	
**Coils**				0.20			0.83			0.37
Bare coils	22	0 (0)	22 (100)		1 (5)	21 (95)		1 (5)	21 (95)	
Modified (Hydrocoil/soft) coils	37	4 (11)	33 (89)		1 (3)	36 (97)		5 (14)	32 (86)	
None	6	0 (0)	6 (100)		0 (0)	6 (100)		0 (0)	6 (100)	
**Occlusion Grade, no. (%)**				0.13			0.13			**0.03**
Class I	23	3 (13)	20 (87)		2 (9)	21(91)		5(22)	18(78)	
Class II	14	1 (7)	13 (93)		0 (0)	14(100)		2(14)	12(86)	
Class III	31	0 (0)	31 (100)		0 (0)	31(100)		0(0)	31(100)	

All *p* value are from Pearson’s x^2^ test or Fisher’s Exact Test (2-sided).

**Figure 1:**
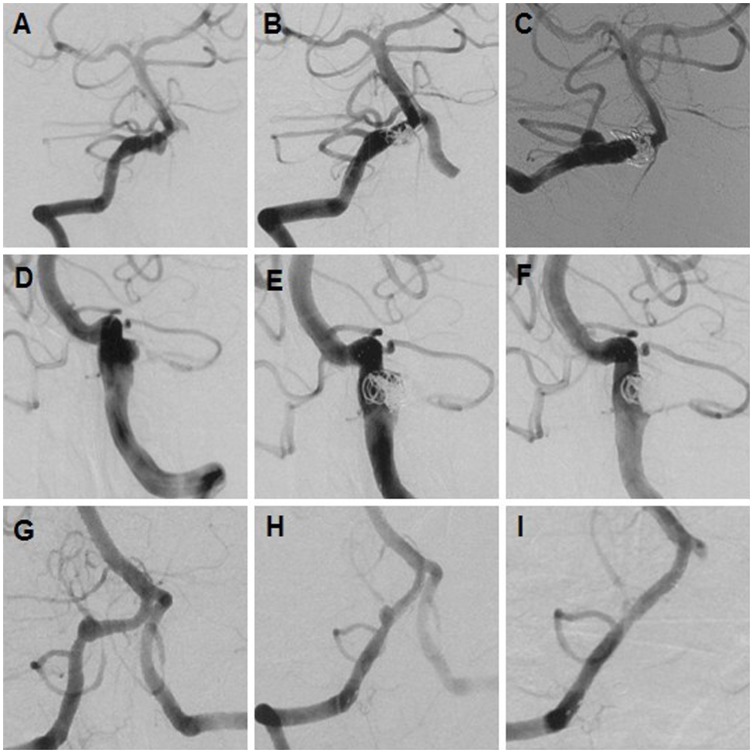
Multiple stents with or without coiling for reconstruction of 3 dissecting aneurysms. (A–C) An involving PICA of VADA (A) recurred (C) 6 months after reconstruction with 3 stents and coils with the immediate partial occlusion (B). (D–F) A cured VADA (D vs F) underwent the treatment of 3 Solitaire stents and coils prior to 3 months with the immediate partial occlusion (E). (G–I) Three Solitaire stent implantations cured a VADA (G vs I) with the straightening curvature of the parent artery (H, I).

Nearly 14% (2/14) of the dissecting aneurysms (2 lateral protrusion-shaped ICADAs) were treated by single stent and coils, and 86% of the dissecting aneurysms (ICADAs, n = 1; VADAs, n = 11) were treated by multiple overlapping stents with (n = 9) or without (n = 3) coils. Although a high rate of class III occlusions (86%, 12/14) was observed in the 14 treated dissecting aneurysms after reconstruction treatment, follow-up angiographic imaging revealed that only 1 (7%) recurred (Fig. 1A–1C), and the remaining 13 (93%) were cured (Table 1). The recurring aneurysm was ultimately resolved by the implantation of an additional Solitaire™ stent.

**Figure 2:**
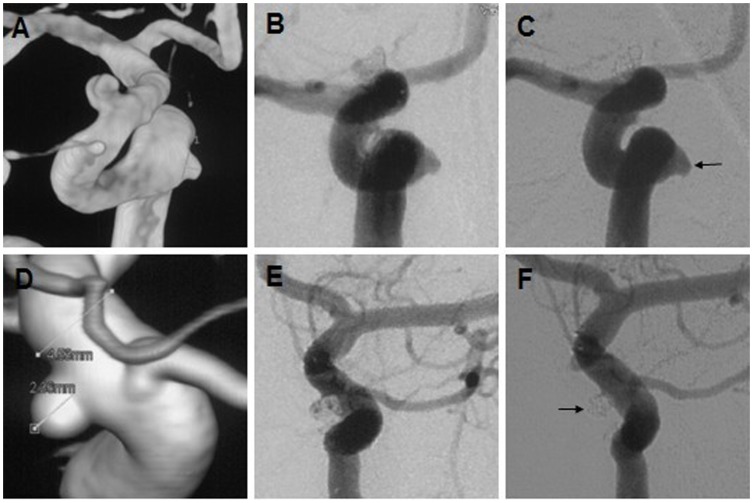
Reconstruction treatment of 2 tiny aneurysms. (A–C) An unruptured tiny aneurysm (A) was reconstructed with a single Solitaire AB stent. The immediate (B) and 12-month (C) angiographic imagings revealed no change with less effective vessel angle modifications. (D–F) An unruptured tiny aneurysm (D) was reconstructed by a single Solitaire stent with coils, achieving immediate residual aneurysm (E). The 12-month (F) angiographic imaging confirmed total occlusion.

Eighteen of the 54 (33%) saccular aneurysms were unruptured and tiny; 3 were reconstructed by single stent implantation (Fig. 2A–2C) and 15 by single stent with coils (Fig. 2D–2F). Of the remaining 36 saccular aneurysms, 33 were treated with a single stent with coils and 3 with 2 overlapping stents and coils. Of these 3, 1 required the implantation of a second Solitaire stent due to acute thrombus formation (Case 1) and the remaining 2 due to coil loop herniation. Upon angiographic imaging during the mean 12-month follow-up period, 3 tiny aneurysms treated with a single stent remained unchanged. Of the remaining 51 saccular lesions, 50 were cured, and 1 improved on the follow-up angiographic imaging.

### Predictors for Adverse Events and Unfavourable Outcomes

**Figure 3:**
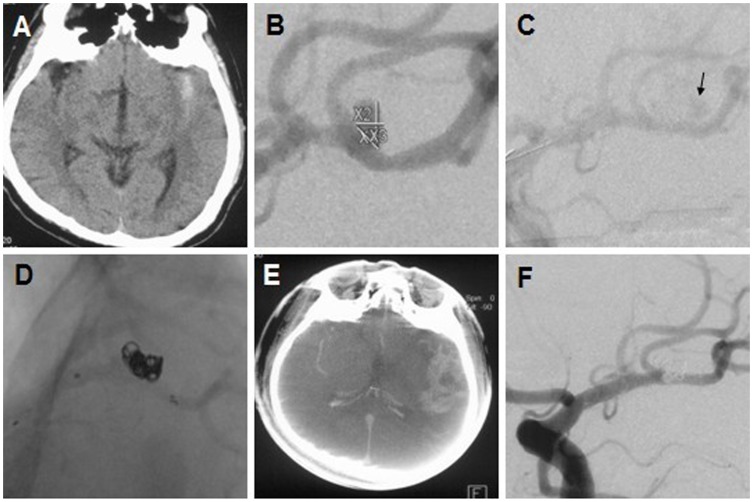
Management of intraprocedural aneurysm rupture (A–B) A ruptured MCA-bifurcation aneurysm. (C) Leakage of contrast medium (arrow) and spasmodism of intracerebral arteries. (D) Morphologic presentation of stent and coils. (E) High density haemorrhage area. (F) The 6-month follow-up angiogram showing the total occlusion of the lesion.

The predictors for outcomes and the management of 6 adverse events are summarised in Tables 3 and 4. All 6 adverse events were encountered during the single stent-assisted coiling for the treatment of tiny (n = 1) or small (n = 5) saccular aneurysms. These adverse events included thromboembolic events (67%, 4/6), whose predictor was SAH (*p<0.05,* Table 2), and aneurysm ruptures (33%, 2/6, Fig. 3). Univariate analysis suggested that the ruptures might be incidental because their risks could not be identified (*p>0.05,* Table 2). In contrast, the predictors for overall adverse events by univariate analysis (Table 2) primarily involved SAH (*p<0.05*) and the occlusion degree (*p<0.05*). The clinical follow-ups of all the patients were available (mean, 19 months; range, 12–27 months), and no rebleeding or new neurological events occurred. SAH (*p<*0.05) was the only predictor for unfavourable outcomes (mRS, 2–6) by univariate analysis (Table 3).

**Table 3:** Effect of Patient Demographics (n = 58) and Aneurysm Angiographic Characteristics (n = 68) on Clinical Outcomes after Endovascular Treatment.

Variables	Favourable Outcomes	Unfavourable Outcomes	Total	*P* Value
**Patient Sex, no. (%)**				*1.00*
Male	27 (96)	1 (4)	28	
Female	28 (93)	2 (7)	30	
**SAH, no. (%)**				**0.004**
Ruptured	9 (75)	3 (25)	12	
Unruptured	56 (100)	0 (0)	56	
**Aneurysm Location, no. (%)**				1.00
Anterior circulation	52 (95)	3 (5)	55	
Posterior circulation	13 (100)	0 (0)	13	
**Aneurysm shape, no. (%)**				1.00
Saccular aneurysm	51 (94)	3 (6)	54	
Dissecting aneurysm	14 (100)	0 (0)	14	
**Aneurysm Size, no. (%)**				0.49
Tiny	18 (100)	0 (0)	18	
Small	44 (94)	3 (6)	47	
Large	3 (100)	0 (0)	3	
**Stent Quantity**				1.00
Single stent with or without coiling	50 (94)	3 (6)	53	
Multiple stents with or without coiling	15 (100)	0 (0)	15	
**Stent Deployment Technique**				0.74
Stent before coil	5 (100)	0 (0)	5	
Stent after coil	54 (95)	3 (5)	57	
Stenting-only	6 (100)	0 (0)	6	
**Coils**				0.33
Bare coils	22 (100)	0 (0)	22	
Modified (Hydrocoil/soft) coils	37 (93)	3 (7)	40	
None	6 (100)	0 (0)	6	
**Occlusion Degree, no. (%)**				0.26
Class I	21 (91)	2 (9)	23	
Class II	13 (93)	1 (7)	14	
Class III (including stenting)	31 (100)	0 (0)	31	

Favourable outcomes (mRS, 0–1); Unfavourable outcomes (mRS, 2–6); All *p* value are from Pearson’s *x* ^2^ test or Fisher’s Exact Test (2-sided).

**Table 4:** Six Patients and the Management of Adverse Events.

No	Age/sex	Presentation	H-H	Locations	Saccular/wide-neck	Size	StentType	Adverse Events	Measures	Occlusion Grade	Follow-upmRS
1	65/F	SAH	II	MCA-bifur	Yes/Yes	Small	ST+ST	Thrombus	Tirofiban+Stenting	Class I	2
2	53/F	Incidental	0	PComA	Yes/Yes	Tiny	ST	Thrombus	Tirofiban	Class I	1
3	56/F	SAH	II	PComA	Yes/Yes	Small	ST	Infarction	No	Class II	2
4	55/M	SAH	IV	AComA	Yes/Yes	Small	ST	Infarction	No	Class I	4
5	48/M	SAH	I	MCA-bifur	Yes/Yes	Small	ST	Rupture	Heparinisation was reversed +total occlusion+ fasudil	Class I	1
6	49/F	Incidental	0	PComA	Yes/Yes	Small	ST	Rupture	Heparinisation was reversed +total occlusion	Class I	1

F, Female; M, Male; SAH, subarachnoid haemorrhage; H-H, Hunt-Hess Scale; MCA, middle cerebral artery; Bifur, Bifurcation; PComA, posterior communicating artery; AComA, the anterior communicating artery; ST, Solitaire.

## Discussion

The striking advances in intracranial stent characteristics [14] and treatment strategies [4], [6], [7] have greatly strengthened the ability to treat complex intracranial aneurysms. Several intracranial stents, such as Neuroform [15] and Enterprise [16], have been well applied in the treatment of intracranial aneurysms. Recently, the Solitaire™ AB (Covidien/ev3) stent was introduced to treat intracranial aneurysms [8], [9]. The Solitaire™ stent has several advantages compared to its competitors. Firstly, the fully retrievable and detachable characteristics make it very easy to accurately deploy the stent. Secondly, the split-shaped design is favourable for the overlapping of the stent within the relatively smaller-sized vessel, resulting in lower stent porosity. In this study, the individualised treatment strategies, including 1–3 Solitaire™ stent(s) with or without coiling, were employed for the treatment of 68 intracranial dissection and saccular aneurysms. To date, this is the largest Solitaire™ AB stent-related sample study involving reconstructive techniques.

The technical success rate of the stent placement was 100%. The rates of the overall and treatment-related adverse events were 9% (6/68) and 1% (1/68), respectively, and the recurrent rate was 1% (1/68). At follow-ups, 94% (64/68) of the lesions and 95% (55/58) of the patients had favourable angiographic and clinical results (Tables 1 and 3), respectively. Progressive thrombosis, in-stent stenosis or occlusion and rebleeding were not encountered. These results indicated the feasibility, safety, and efficacy of individualised treatments with the Solitaire stent.

The majority (86%, 12/14) of the dissecting aneurysms were reconstructed using multiple stents with (n = 9) or without (n = 3) coils for the treatment of particular anatomic characteristics, such as lesions with wide-necked lateral protrusion-shaped configurations, fusiform aneurysms without defined necks, PICA-involving lesions, and lesions originating from the artery which has predominant blood supply to the basilar artery. Furthermore, the ruptured dissection aneurysms were all treated with multiple stents and coils due to concerns of rebleeding. In contrast, all the treated small saccular aneurysms, including the tiny ones, were treated due to one of the following reasons: (1) ruptured lesions; (2) symptomatic lesions; (3) anxiety of lesion ruptures by patients with unruptured small or tiny aneurysms (Table 4, Case 2). Despite the development of class III occlusions in 86% of the patients (12/14) receiving 1–3 stent(s) with or without coils, the follow-up angiographic results revealed that 13 were cured and only 1 recurred (Table 1). This may be attributed to several stent-related advantages: (1) “scaffolding” for denser packing [17], primarily involving stenting prior to coiling (mesh technique) and stenting after coiling (jailing or semi-jailing [18]) (Table 2); (2) reconstruction of the diseased parent artery by “endovascular bypass”; (3) the flow diversion or stent-associated flow remodelling [19], resulting in further occlusion of incompletely coiled aneurysms (Fig. 1E–1F); (4) the effective vascular angle modification (Fig. 1G–1I); (5) reattachment of the dissected arterial tissue flap and closing off of the false lumen. One dissecting aneurysm involved the origin of PICA. This aneurysm recurred after treatment with 3 overlapping stents and coils with immediate partial obliteration. An additional Solitaire stent implantation ultimately cured this recurrent lesion, confirming that the relatively lower stent porosity was crucial for preventing the recurrence. This corresponded with an experimental study where hemodynamic modifications were proportional to the number of overlapping stents [20]. In the future, newly designed stents with low porosity, such as the SILK flow diverter [21], may help to overcome the limitations of a high-porosity stent and improve the treatment of saccular and fusiform aneurysms [21], [22], [23]. Another alternative explanation for the recurrence was that the curvature of the parent vessels bearing the aneurysms were not effectively modified (Fig. 1A–1B). Such modifications are crucial for angiographic outcomes [24], [25], as was demonstrated in Fig. 1G–1I.

The majority (94%, 51/54) of the saccular aneurysms were treated with a single stent with (n = 48) or without (n = 3) coils. However, 3 cases were treated with 2 overlapping stents, one of which was treated with a second Solitaire stent implantation for acute thrombus formation (Case 1, Table 4), and the remaining 2 were treated due to coil loop herniation. These 3 cases were all treated with eventual favourable outcomes. Angiographic imaging at the mean 12-month follow-ups revealed that the 3 sole stenting tiny aneurysms remained unchanged. The delayed occlusion time may have been related to less effective angle corrections of the parent vessels (Fig. 2A–2C). Although the class III occlusion rate was up to 35% in the 54 saccular aneurysms (19/54), follow-up imaging revealed that 50 were cured (93%), 1 was improved (2%), and 3 were unchanged (5%). Moreover, post-treatment rebleeding was not encountered. These results support the view that stent implantation can lead to further occlusion of incompletely coiled aneurysms and prevent haemorrhage [15], [17], [19], [26].

Stent implantation is regarded as a thrombogenic risk up until stent endothelialisation [27]. One concern is whether the increased quantity of embedded stent(s) increases the risks of thromboembolic events. However, this series of 4 thromboembolic events all occurred in single-stent-assisted coiling, 3 of which occurred in SAH patients. The univariate analysis showed that the predictor for thromboembolic events was not the quantity of embedded stents (*p>0.05*), but SAH (*p<0.05,* Table 2), which supports the idea that use of adjunctive devices in treating aneurysms does not increase the frequency of embolic or ischemic events [28]. The occurrence of these adverse events may be related to several causes, such as the relatively unfavourable anatomic access, the lack of experience in the early stage of treatment, the SAH-related hypercoagulable state [29], vasospasm, and the relatively short duration of antiplatelet therapy prior to stent implantation. Additionally, we eliminated two thrombus events by the infusion of sole tirofiban (Case 2) and the combination of tirofiban and a second Solitaire stent implantation (Case 1). In contrast, postprocedural infarction events remained inevitable (Case 3–4), particularly in SAH patients, which required further investigation. In addition to SAH, the occlusion grade was also a predictor for overall adverse events (*p<0.05*, Table 2). The rate of up to 96% (65/68) in tiny (n = 18) and small (n = 47) lesions might be responsible for this predictor.

In the early stage of treatment, 2 intraprocedural aneurysm ruptures were encountered, whose risk factors could not be identified (*p>0.05,* Table 2) due to the limited data. The pre-deployed stent enabled the rapid occlusion of ruptured lesions, preventing severe bleeding (Fig. 3A vs Fig. 3E). There were two independent cases that had mRS scores of 1 at discharge and at follow-ups (Case 5–6). These 2 small ruptured lesions were located on the posterior communicating artery (PComA) and the middle cerebral artery (MCA) bifurcation, which supports the viewpoint that the rate of intraoperative rupture is related to aneurysm size and location [30]–[31].

Another principal finding was that SAH was the only predictor for unfavourable outcomes (mRS, 2–6) by univariate analysis (*p<*0.05, Table 3). This suggests that SAH is involved in the outcomes of unruptured [10] or ruptured [32] vertebrobasilar artery dissections, but it appears to be discordant with the role of SAH in saccular aneurysm outcomes [33]. This may be explained by differing definitions of clinical outcomes in a study by Tähtinen et. al. where good clinical outcome was defined by a Glasgow Outcome Scales (GOS) score of 5, moderate outcome by a GOS score of 3 or 4, and poor outcome by a GOS score of 1 or 2 [33].

### Conclusions

Although the complete obliteration of tiny and small wide-necked aneurysms without complications remains a technical challenge, stent implantation can lead to further occlusion of incompletely coiled aneurysms. The predictors for adverse events primarily involved SAH and the occlusion grade. SAH was the only predictor for unfavourable outcomes by univariate analysis.
